# The English and German Physician through French Eyes

**Published:** 1910-04-23

**Authors:** 


### The English and German Physician through French Eyes.

To medical men it is always interesting to note the impression made by our native practitioners upon a foreign colleague, and the interest is increased when an opportunity occurs of comparing this impression with that made on the same individual by practitioners of a third country. It is therefore without apology that the following somewhat free translation is given of portions of an article which recently appeared in *L'Union Médicale du Nord-Est*, from the pen of Dr. Colleville, a hospital physician of Rheims. The author would seem to have had the opportunity of studying at first hand both the English and the German physician, chiefly of the consulting class, his remarks having only occasional application so far as the general practitioner of this country, at any rate, is concerned. He observes that "in England the physician, coming as a rule from a family in easy circumstances, finds himself sheltered from want and removed from the fierce competition and incessant struggle to 'arrive.' The friend of the family and its adviser in many things extra-medical, the English doctor directs his patients towards the specialist as often as this is necessary. Without professional cares he lives in his sphere between his home and his country seat, and he knows the joys of holidays. . . . As a student he has respect for the University which gives him his diploma, and as a young man he likes to leave at his *alma mater* the remembrance of all the prizes earned by his profound experience in sport. Later on, when celebrated, a statue will be erected to him, and his bust will show future generations *inter prandia et inter preces* that the 'right man' must at the same time have the qualities of a Porthos and an Aramis. He simplifies his bookkeeping by taking payment on the spot for each visit and each consultation. Social medicine has its State diplomas obtained as the result of examinations in which public hygiene holds first place. This service is sufficiently well paid to allow private practice to be dispensed with. The title of Fellow of several royal societies is the highest rank to which the savant across the Channel can attain. The numerous anatomical preparations of the Brothers Hunter and their disciples, and the experiments of the School of Horsley show that the study of anatomy and physiology is in great vogue among our neighbours."

The German physician, on the other hand, knows the effects of competition, for, according to Dr. Colleville, he has to fight unqualified empiricists, since in Germany the exercise of medicine is unfettered, and medical functionaries poorly paid by the numerous and powerful companies of assurance against disease and invalidity. There also flourishes on Teuton soil *propter necessitatem* the Deutsche Aerztevereinsband or great central union of associations to defend material and moral interests. The members of provincial medical chambers do not remain idle. In controversial cases the assent to decrees is left in the hands of public magistrates. "The State, which confers all diplomas, is anxious for the good repute of its scientists. According to their seniority in the practice of medicine and to the value of their scientific work they are honoured with the title of Councillors of State . . . and even with that of professors, although they do not always belong to a teaching body. The man of merit is bargained for by the Universities. He is no longer, as in England, a matter for college attention, but a piece of national luxury. Given the extraordinary aptitude which the German possesses for commerce, the best man in his region creates for himself a reputation, of which the Emperor knows how to make the best use should occasion arise. If in England the laboratory ministers to suffering humanity with the ultimate object of diminishing the inroads upon the budget of society, in Germany the study of science is intensified with a double object—economy and patriotism. *Ad majorem Germaniæ gloriam.*"

